# Specialized healthcare diagnostic events within one year preceding colorectal cancer diagnosis

**DOI:** 10.2340/1651-226X.2026.44788

**Published:** 2026-03-01

**Authors:** Elina M. Hermiö, Salla E. Toikkanen, Liisa H. Pylkkänen, Jutta Järvelin, Janne M. Pitkäniemi, Nea K. Malila, Karri J. Seppä

**Affiliations:** a Finnish Cancer Registry, Helsinki, Finland; b Faculty of Social Sciences, Health Sciences, Tampere University, Tampere, Finland; c Department of Oncology, University of Turku, Turku, Finland; d Finnish Institute for Health and Welfare, Helsinki, Finland; e Department of Public Health, Faculty of Medicine, University of Helsinki, Helsinki, Finland

**Keywords:** health services, specialized care, colorectal cancer, health services utilization

## Introduction

Colorectal cancer (CRC) is the second most common cancer in Finland and the burden of CRC is increasing worldwide [1–3]. The majority of CRC cases are diagnosed at an older age or at advanced stages due in part to non-specific symptoms [1, 4–6].

The use of health services (HS) starts to increase a few months before CRC diagnosis [7, 8]. Several studies [9–11] have reported pre-diagnostic symptoms, clinical features and prescribed medicine before CRC diagnosis [12, 13]. However, few have described diagnostic groups for healthcare utilization.

Our aim was to assess the occurrence of HS events in specialized healthcare settings by diagnostic codes during the year preceding CRC diagnosis.

## Patients/material and methods

Incident CRC cases with International Classification of Diseases and Related Health Problems, 10^th^ revision (ICD-10) codes C18-C20 [14] diagnosed in 2015 were retrieved from the Finnish Cancer Registry (FCR) [15, 16]. All HS events (outpatient visits or inpatient stays in specialized HS) within 1 year before CRC diagnosis were retrieved from the Care Register for Health Care (Hilmo) [17].

Sex, date of diagnosis, stage, and age of patient at the time of CRC diagnosis were available from the FCR, and the dates of HS events with diagnostic ICD-10-codes from Hilmo. The individual data from FCR and Hilmo were linked using the unique personal identity number.

Diagnostic codes for HS events were classified into diagnostic ICD‑10 groups according to the first character of each code, forming homogeneous groups that represent diseases with shared characteristics or a single defined condition [14] (Table 1). We focus on ICD-10 diagnostic groups with at least one visit by 15% or more of the patients: Malignant neoplasms (C), Neoplasms and diseases of the blood (D), Diseases of the digestive system (K), Symptoms, signs and abnormal clinical and laboratory findings (R), and No events within 1 year before diagnosis (No events). Codes considered not relevant for CRC were grouped into one category, ‘Other diagnoses’. Furthermore, codes Diseases of oral cavity, salivary glands and jaws (K00–K14); Injuries, poisoning, and certain other consequences of external causes (S00–T98); External causes of morbidity (V00–Y99) and other non-specific factors influencing health status and contact with HS (Z00–Z99) were not included (Table 1).

**Table 1:** Number of HS events and number and proportion of colorectal cancer patients (C18–C20) having at least one HS event within 1 year preceding CRC diagnosis by sex and age-adjusted relative risk for women versus men (RR) with 95% credible intervals by diagnostic group.*

Diagnostic group	Men			Women			Relative Risk^1^	95% CI
	Number of HS events	Number of patients	Proportion (%)^1^	Number of HS events	Number of patients	Proportion (%)^1^		
Certain infectious and parasitic diseases (A)	153	67	4.09	87	55	3.63	0.90	0.62–1.26
Certain infectious and parasitic diseases (B)	20	7	0.39	5	4	0.29	0.86	0.18–2.38
Malignant neoplasms (C)	1660	320	19.05	927	247	16.98	0.89	0.75–1.05
Neoplasms and diseases of the blood (D)	839	389	23.35	905	440	30.10	1.29	1.12–1.48
Endocrine, nutritional and metabolic diseases (E)	144	66	3.97	92	37	2.49	0.64	0.41–0.93
Mental and behavioural disorders (F)	115	38	2.33	114	27	1.83	0.81	0.47–1.28
Diseases of the nervous system (G)	196	94	5.51	152	67	4.65	0.85	0.61–1.15
Diseases of the eye and ear (H)	445	164	9.92	425	168	11.34	1.15	0.92–1.42
Diseases of the circulatory system (I)	698	293	17.59	435	203	13.57	0.77	0.64–0.92
Diseases of the respiratory system (J)	232	114	6.86	178	87	5.93	0.87	0.65–1.15
Diseases of the digestive system (K)	99	353	21.41	662	306	20.74	0.97	0.83–1.13
Diseases of the skin (L)	106	30	1.80	65	36	2.38	1.37	0.81–2.19
Diseases of the musculoskeletal system (M)	286	103	6.02	294	122	8.53	1.43	1.09–1.85
Diseases of the genitourinary system (N)	414	109	6.48	229	110	7.56	1.17	0.89–1.52
Symptoms, signs and abnormal clinical and laboratory findings (R)	511	277	16.61	420	238	16.31	0.98	0.82–1.16
Pregnancy, childbirth (O); Conditions in the perinatal period (P); Congenital malformations (Q); Codes for special purposes (U), (Other diagnoses)	25	14	0.82	16	7	0.45	0.60	0.20–1.32
No pre-diagnosis events (No events)	498	498	29.22	391	391	26.99	0.93	0.81–1.05
Total^2^	6643	1682		5006	1461			

1 Age-adjusted proportion of patients and relative risk (RR) with 95% credible intervals.

2 Total number events does not include ‘No events’.

* Codes concerning Diseases of oral cavity, salivary glands and jaws (K00–K14); Injuries, poisoning, and certain other consequences of external causes (S00–T98); External causes of morbidity (V00–Y99) and other non-specific factors influencing health status and contact with health services (Z00–Z99) were not included. HS: health services; CRC: colorectal cancer; CI: confidence interval.

Stage of cancer was classified into three levels: localized (including localized and locally advanced, i.e. any T and N0 M0), non-localized (including both regional lymph node metastases and distant metastases, i.e. *N* or *M* ≠ 0), and unknown (no information on stage of cancer). For age at diagnosis, age groups under 65 years, 65–74 years, 75–84 years, and 85 years or over were used.

### Statistical methods

The binary outcome was a patient having at least one HS event with a specific diagnostic ICD-10 group within 1 year preceding CRC diagnosis. We estimated the proportion of patients by diagnostic group. The respective proportions were modelled using Bayesian Poisson regression with the total number of patients as offset and standardized by age and sex using the number of patients in the four age groups by sex as weights. The standardized proportions were compared by estimating patients’ relative risks (RR) and their 95% confidence intervals (CI), using men, the youngest age group, or localized stage as the reference categories. Statistical analyses were performed using R software version 4.4.1 and rjags -package [18, 19].

This study was approved by the Finnish Institute for Health and Welfare (THL) (THL/201/6.02.00/2016).

## Results

In 2015, 3143 CRC patients were diagnosed in Finland (54% men, 46% women; median age 71, range 22–96). Cancer stage was localized in 32%, non-localized in 42%, and unknown in 26%. During the year preceding diagnosis, 11,649 HS events were recorded. However, 889 (28%) patients had no preceding HS events recorded (Table 1 and Supplementary Table 1).

The largest proportions of patients with at least one HS event were in diagnostic categories: Neoplasms and diseases of the blood (group D, 26%), Diseases of the digestive system (group K, 21%), Malignant neoplasms (group C, 18%), and Symptoms, signs and abnormal clinical and laboratory findings (group R, 16%).

The age-standardized proportion of patients with at least one HS event in diagnostic group D (Neoplasms and diseases of the blood) was larger in female patients than in male patients (30% vs. 23%, RR 1.29, 95% CI 1.12–1.48) and in diagnostic group I (Diseases of the circulatory system) the proportion of female patients was smaller than in male patients (14% vs. 18%, RR 0.77, 0.64–0.92) (Supplementary Figure 1).

In diagnostic groups C Malignant neoplasms (20% vs. 13%), D Neoplasms and diseases of the blood (31% vs. 23%), and K Diseases of the digestive system (27% vs. 22%), the proportion of patients was larger in the oldest compared with the youngest age group, respectively. In group ‘No events’, the proportion of patients was smaller in the oldest age group compared with the youngest age group (18% vs. 36%) (Supplementary Figure 2)

The most frequent diagnosis in group D (Neoplasms and diseases of the blood) was anaemia (ICD-10 codes D50.9, D64.9 and D50.0) present in 45% of patients with at least one event in group D. Benign neoplasm of colon, rectum, anus and anal canal (D12.0, D12.2, D12.3, D12.5, D12.6. D12.7 and D12.8) occurred in 29%, and neoplasm of uncertain behavior of oral cavity and digestive organs (D37.4 and D37.5) occurred in 22% of patients, respectively.

CRC patients with at least one group K event (Diseases of the digestive system), intestinal obstruction (K56.6; 12% of patients), melena (K92.1; 9%, respectively), and rectal bleeding (K62.5; 8%, respectively) were most common diagnoses. Respectively, in group R (Symptoms, signs and abnormal clinical and laboratory findings), stomach or lower abdominal pain (R10.3, R10.4) occurred in 36% of patients. In group C (malignant neoplasms), CRC (C18–C20) was the most common diagnosis, affecting 42% of patients with at least one group C event.

**Figure 1:**
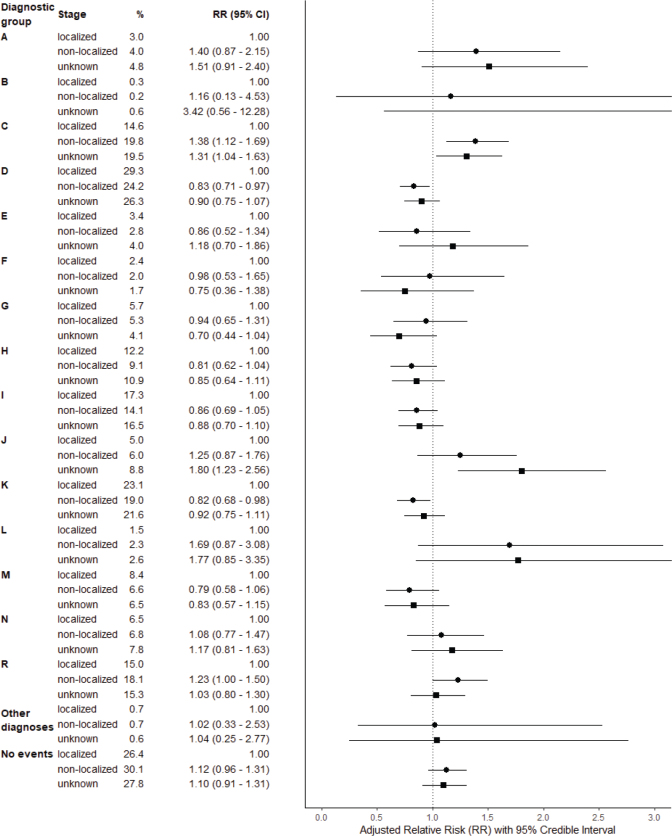
Proportion of colorectal cancer patients having at least one HS event within 1 year preceding CRC diagnosis and age and sex adjusted relative risk (RR) with 95% credible intervals by diagnostic group of HS event and stage. (Foot note: localized stage as reference category). HS: health services; CRC: colorectal cancer.

The proportion of patients having at least one HS event in diagnostic group D (Neoplasms and diseases of blood) was smaller in non-localized than in localized CRC (24% vs. 29%; RR 0.83, 0.71–0.97). In contrast, at least one HS event in diagnostic group C (Malignant neoplasms) was more common among patients with non-localized compared with localized CRC (20% vs. 15%, RR 1.38, 1.12–1.69). The proportion of patients with at least one HS event in diagnostic group K (Diseases of the digestive system) was smaller in non-localized than in localized CRC (19% vs. 23%, RR 0.82, 0.68–0.98), whereas in diagnostic group R, the proportion was larger among patients with non-localized than in localized CRC (18% vs. 15%, RR 1.23, 1.00–1.50) (Figure 1).

## Discussion and conclusion

We found the proportion of patients with specialized HS events to be predominant in diagnostic groups Neoplasms and diseases of the blood (D), Diseases of the digestive system (K), Malignant neoplasms (C), and Symptoms, signs and abnormal clinical and laboratory findings (R), all of which can be linked to the diagnostic process of CRC [5, 12, 13, 20–22]. Surprisingly, a considerable proportion of patients (28%) had no pre-diagnostic HS events within 1 year before diagnosis, with the highest proportion observed among the youngest age group.

After adjusting for age and sex, patients with non-localized or unknown-stage CRC were more likely to have no pre-diagnostic HS events compared to those with localized disease. The finding suggests that some patients with non-localized cancer were admitted urgently with no recorded HS events in the year preceding CRC diagnosis [5]. Our findings are consistent with prior research showing that a significant proportion of inpatient stays within 1 year before CRC diagnosis started as urgent admissions [7] and that 16% of cancers were diagnosed via unplanned hospital admissions [23]. Furthermore, the presence of non-specific symptoms can contribute to delays in CRC diagnosis in both patients and clinicians [12].

In diagnostic group C, the proportion of patients was larger in the non-localized and unknown stages compared to localized stage, likely reflecting the diagnosis code given before confirmation of diagnosis. The lower HS event risk observed for diagnostic groups D and K among non‑localized and unknown cases suggests that symptoms were either too non‑specific to prompt care seeking or were absent [12]. Also other factors, such as understanding the need for medical care, willingness to receive care, mistrust of healthcare organizations, and access to HS, may have influenced the use of HS in patients with non-localized cancer [24–26].

This register-based study avoided recall bias as it was based on comprehensive data from national health registries [15, 27]. A key strength was the ability to examine pre-diagnostic HS events in CRC patients with latent disease while preparing for national screening policy [28]. The main limitations included missing data on primary HS, which may lead to an underestimation of pre-diagnostic HS events.

With our data, we were able to identify specialized HS diagnoses that can be utilized for earlier detection of patients. These data are critical for characterizing diagnostic trajectories and understanding healthcare organization, enabling identification of patterns that may inform strategies for early detection and improved care pathways.

## Supplementary Material



## Data Availability

The data that support the findings of this study can be requested in aggregate form from (FCR), but they are not publicly available due to data secrecy issues.
