# Supplementary material for: Specialized healthcare diagnostic events within one year preceding colorectal cancer diagnosis
Source: Acta Oncol. 2026 Mar 1;65:44788. doi: 10.2340/1651-226X.2026.44788 (PMC12961377; doi:10.2340/1651-226X.2026.44788)

Supplementary material has been published as submitted. It has not been copyedited, or typeset by Acta Oncologica

Supplementary Table 1: Number and proportion of colorectal cancer patients (C18-C20) by sex, age groups and stage of cancer within a year before colorectal cancer diagnosis.

		MEN	PROPORTION OF MEN (%)	WOMEN	PROPORTION OF WOMEN (%)	TOTAL
**AGE GROUPS**	Under 65	449	26,7	403	27,6	**852**
	65-74	585	34,8	425	29,1	**1010**
	75-84	490	29,1	398	27,2	**888**
	85+	158	9,4	235	16,1	**393**
	**Total**	**1682**	**100,0**	**1461**	**100,0**	**3143**
**STAGE**	Localized	535	31,8	474	32,4	**1009**
	Non-localized	710	42,2	614	42,0	**1324**
	Unknown	437	26,0	373	25,5	**810**
	**Total**	**1682**	**100,0**	**1461**	**100,0**	**3143**

Supplementary Figure 1: Proportion of colorectal cancer patients having at least one HS event within one year preceding CRC diagnosis and age adjusted relative risk (RR) with 95% credible intervals by diagnostic group of HS event and sex. (Foot note: men as a reference category.)

Diagnostic group	Sex	%	RR (95% CI)
**A**	male	4.0	1.00
	female	3.8	0.90 (0.62 - 1.26)
**B**	male	0.4	1.00
	female	0.3	0.86 (0.18 - 2.38)
**C**	male	19.0	1.00
	female	16.9	0.89 (0.75 - 1.05)
**D**	male	23.1	1.00
	female	30.1	1.29 (1.12 - 1.48)
**E**	male	3.9	1.00
	female	2.5	0.64 (0.41 - 0.93)
**F**	male	2.3	1.00
	female	1.8	0.81 (0.47 - 1.28)
**G**	male	5.6	1.00
	female	4.6	0.85 (0.61 - 1.15)
**H**	male	9.8	1.00
	female	11.5	1.15 (0.92 - 1.42)
**I**	male	17.4	1.00
	female	13.9	0.77 (0.64 - 0.92)
**J**	male	6.8	1.00
	female	6.0	0.87 (0.65 - 1.15)
**K**	male	21.0	1.00
	female	20.9	0.97 (0.83 - 1.13)
**L**	male	1.8	1.00
	female	2.5	1.37 (0.81 - 2.19)
**M**	male	6.1	1.00
	female	8.4	1.43 (1.09 - 1.85)
**N**	male	6.5	1.00
	female	7.5	1.17 (0.89 - 1.52)
**R**	male	16.5	1.00
	female	16.3	0.98 (0.82 - 1.16)
**Other diagnoses**	male	0.8	1.00
	female	0.5	0.60 (0.20 - 1.32)
**No events**	male	29.6	1.00
	female	26.8	0.93 (0.81 - 1.05)

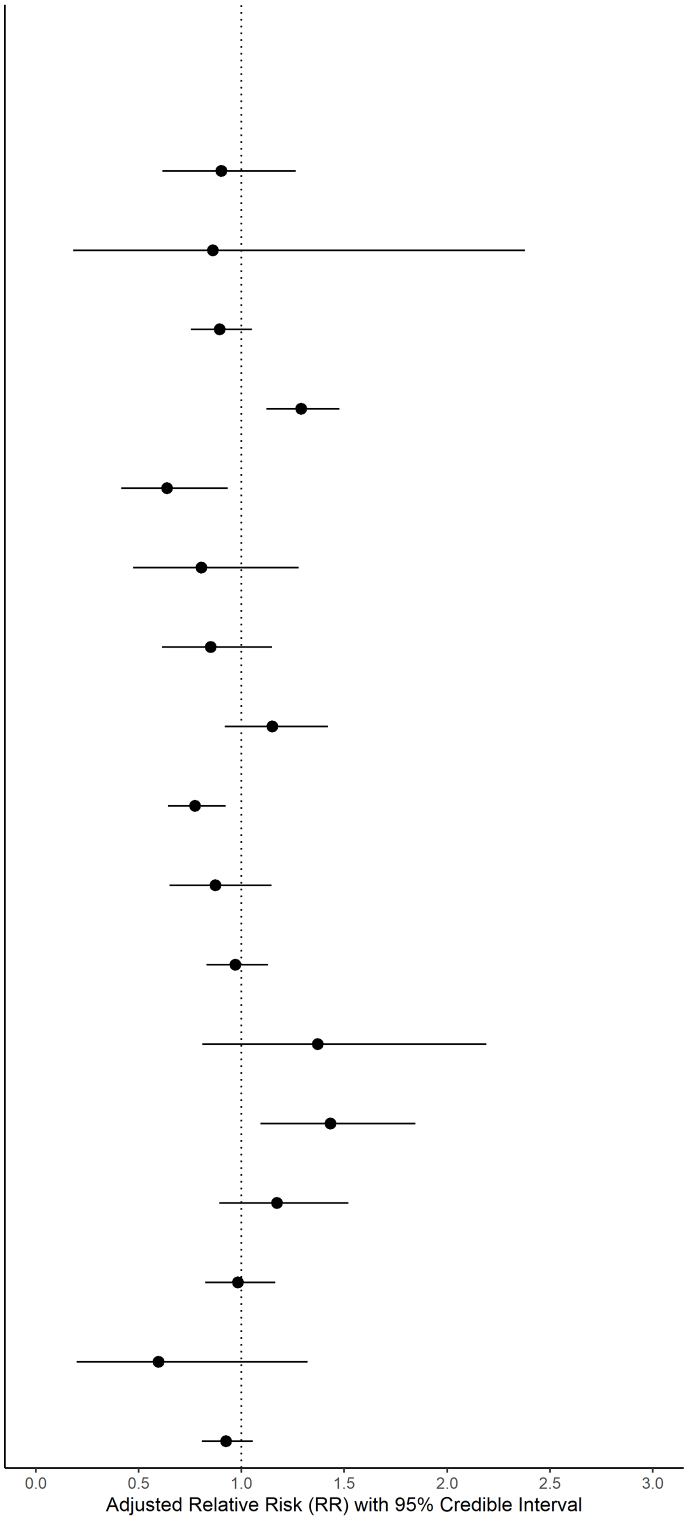

Supplementary Figure 2: Proportion of colorectal cancer patients having at least one HS event within one year preceding CRC diagnosis and sex adjusted relative risk (RR) with 95% credible intervals by diagnostic group of HS event and age. (Foot note: age group 65 years and under as a reference category.)

Diagnostic group	Age group	%	RR (95% CI)
A	<65	2.7	1.00
	65-74	2.8	1.07 (0.59 - 1.82)
	75-84	5.0	1.91 (1.12 - 3.12)
	85+	6.9	2.67 (1.45 - 4.53)
B	<65	0.1	1.00
	65-74	0.6	46.90 (0.93 - 171.03)
	75-84	0.5	38.93 (0.64 - 143.85)
	85+	0.0	0.32 (0.00 - 0.64)
C	<65	12.6	1.00
	65-74	20.2	1.61 (1.27 - 2.03)
	75-84	20.0	1.61 (1.26 - 2.03)
	85+	19.8	1.67 (1.22 - 2.23)
D	<65	22.8	1.00
	65-74	25.0	1.12 (0.93 - 1.35)
	75-84	29.2	1.30 (1.07 - 1.56)
	85+	31.3	1.38 (1.09 - 1.73)
E	<65	3.4	1.00
	65-74	3.2	0.94 (0.55 - 1.51)
	75-84	2.9	0.88 (0.50 - 1.45)
	85+	4.1	1.30 (0.65 - 2.29)
F	<65	1.5	1.00
	65-74	1.9	1.35 (0.63 - 2.64)
	75-84	2.3	1.58 (0.74 - 3.05)
	85+	3.3	2.53 (1.04 - 5.18)
G	<65	4.3	1.00
	65-74	5.3	1.27 (0.81 - 1.89)
	75-84	6.3	1.49 (0.96 - 2.21)
	85+	3.6	0.89 (0.44 - 1.58)
H	<65	4.0	1.00
	65-74	9.3	2.44 (1.62 - 3.58)
	75-84	15.4	3.99 (2.70 - 5.77)
	85+	17.0	4.37 (2.83 - 6.55)
I	<65	8.3	1.00
	65-74	13.5	1.62 (1.21 - 2.14)
	75-84	22.1	2.67 (2.02 - 3.48)
	85+	23.7	2.88 (2.07 - 3.89)
J	<65	4.9	1.00
	65-74	6.4	1.32 (0.88 - 1.91)
	75-84	7.1	1.48 (0.98 - 2.15)
	85+	7.9	1.74 (1.04 - 2.72)
K	<65	21.6	1.00
	65-74	18.2	0.84 (0.68 - 1.03)
	75-84	20.7	0.97 (0.78 - 1.18)
	85+	27.2	1.32 (1.02 - 1.67)
L	<65	1.4	1.00
	65-74	2.2	1.70 (0.79 - 3.29)
	75-84	1.9	1.49 (0.67 - 2.95)
	85+	3.8	2.74 (1.18 - 5.59)
M	<65	6.6	1.00
	65-74	7.2	1.14 (0.79 - 1.59)
	75-84	9.1	1.43 (1.01 - 1.99)
	85+	3.8	0.57 (0.29 - 0.96)
N	<65	5.5	1.00
	65-74	7.1	1.34 (0.91 - 1.91)
	75-84	8.1	1.51 (1.03 - 2.15)
	85+	7.1	1.37 (0.82 - 2.14)
R	<65	14.8	1.00
	65-74	16.1	1.10 (0.87 - 1.38)
	75-84	17.2	1.17 (0.92 - 1.48)
	85+	18.6	1.31 (0.96 - 1.74)
Other diagnoses	<65	0.8	1.00
	65-74	0.8	1.07 (0.33 - 2.68)
	75-84	0.3	0.47 (0.07 - 1.39)
	85+	0.8	1.04 (0.16 - 3.26)
No events	<65	36.4	1.00
	65-74	30.4	0.83 (0.71 - 0.97)
	75-84	22.6	0.62 (0.52 - 0.74)
	85+	18.1	0.49 (0.37 - 0.63)

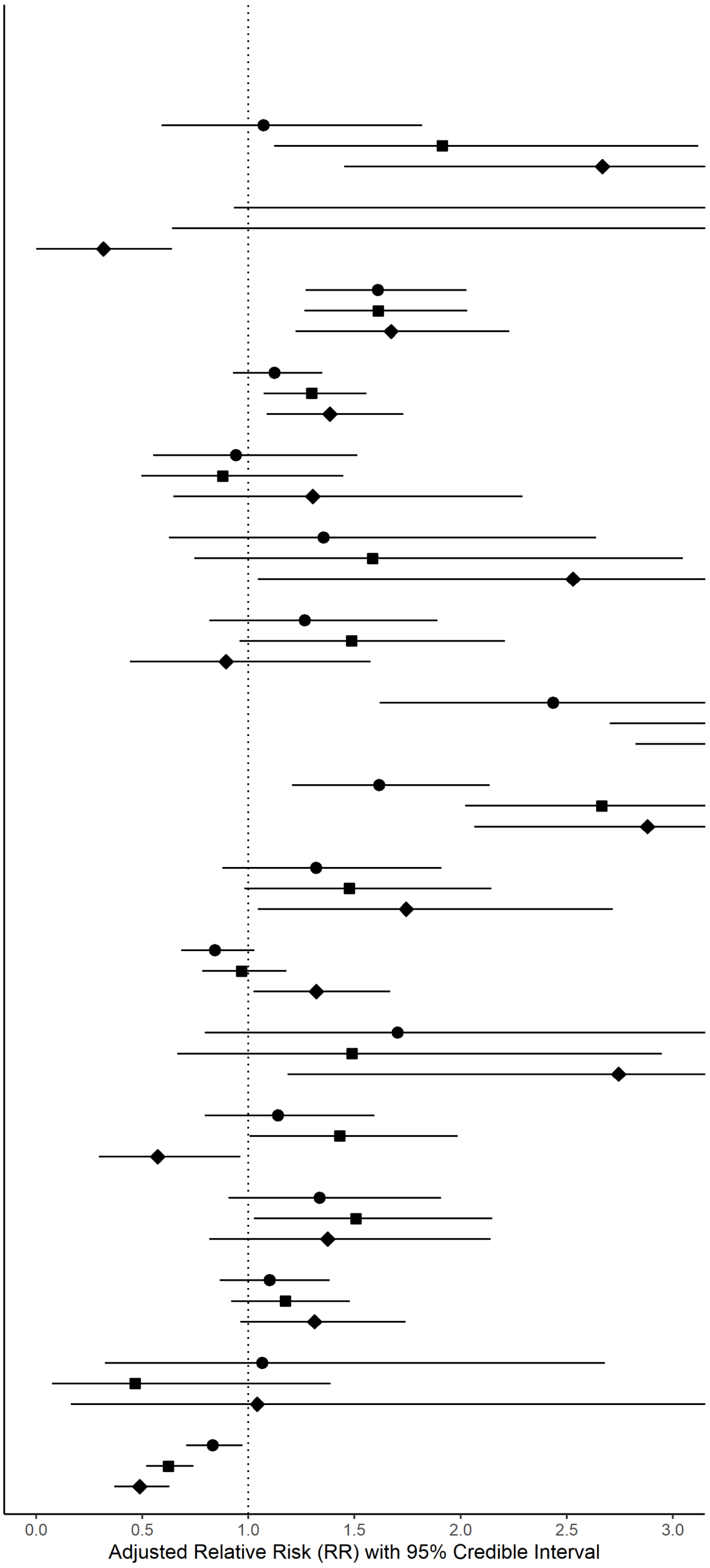

Supplement: Supplementary file 1 [file AO-65-44788-s1.pdf]
